# Three controversies over item disclosure in medical licensure examinations

**DOI:** 10.3402/meo.v20.28821

**Published:** 2015-09-14

**Authors:** Yoon Soo Park, Eunbae B. Yang

**Affiliations:** 1 Department of Medical Education, University of Illinois College of Medicine, Chicago, IL, USA; 2 Department of Medical Education, Yonsei University College of Medicine, Seoul, South Korea

**Keywords:** fairness, validity, utility of disclosure, passing level, standards in testing

## Abstract

In response to views on public's right to know, there is growing attention to *item disclosure* – release of items, answer keys, and performance data to the public – in medical licensure examinations and their potential impact on the test's ability to measure competence and select qualified candidates. Recent debates on this issue have sparked legislative action internationally, including South Korea, with prior discussions among North American countries dating over three decades. The purpose of this study is to identify and analyze three issues associated with item disclosure in medical licensure examinations – 1) fairness and validity, 2) impact on passing levels, and 3) utility of item disclosure – by synthesizing existing literature in relation to standards in testing. Historically, the controversy over item disclosure has centered on fairness and validity. Proponents of item disclosure stress test takers’ right to know, while opponents argue from a validity perspective. Item disclosure may bias item characteristics, such as difficulty and discrimination, and has consequences on setting passing levels. To date, there has been limited research on the utility of item disclosure for large scale testing. These issues requires ongoing and careful consideration.

There is a growing public interest on identifying the impact of item disclosure on medical licensure examinations. *Item disclosure* refers to publicly releasing test items, answer keys, and performance data, in response to views on the public's right to know and fairness. The debate on the effect of item disclosure has a long history in North America dating to the 1970s; the Committee on Ability Testing of the National Academy of Sciences in the United States launched a study on item disclosure in 1978, and since then, there have been several studies on the utility of disclosure. For example, there have been attempts to examine the influence of item disclosure on test and item characteristics, including item difficulty, impact on reusing disclosed test items, and consequences on passing levels. Studies have presented diverse and competing conclusions from several perspectives, including legal scholars, education researchers, and statisticians (1–5). Overall, these discussions have asserted that item disclosure can bias measurement of test taker performance, while others have found limited evidence for such an association.

Item disclosure has many important implications. While item disclosure may satisfy examinees’ right to know, help test takers prepare for the exam, and increase public transparency in test administration for licensure examinations, it could affect the maintenance and management of large item banks in testing organizations, raise test difficulty, alter appropriate passing levels, and increase test development and administrative costs. Medical license examinations are high-stakes examinations, which serve to screen candidates and ensure that they are equipped with the required knowledge, skills, and attitude for practice. However, to date, calls for item disclosure have continued, with new international perspectives joining the discussion. For example, some countries, such as South Korea, have recently established a fixed passing score of 60%. In such countries, investigating the influence of item disclosure holds even greater importance.

Despite increasing attention, a critical analysis on the effects of item disclosure that synthesizes existing discussion, within the context of international testing standards, is needed (6). The potential influence of disclosure on ways of developing and selecting test items should be examined. Many countries have adopted item banking systems to maintain the validity and reliability of licensure exams. If test items are routinely disclosed to the public, there will be a need to identify whether such item banking systems can be maintained and what changes might be needed after each cycle of item disclosure.

Against this backdrop, we analyze three issues related to item disclosure: 1) fairness and validity, 2) impact on passing levels, and 3) utility of item disclosure. This paper provides a historic overview of item disclosure and a synthesis of existing literature within the context of international testing standards to provide implications of item disclosure for medical licensing examinations. Given that item disclosure is facing increased attention internationally, our goal is to provide meaningful insights for countries and agencies debating the consequences of item disclosure.

## Fairness and logic issues in the disclosure of licensure examination items

### Conflicting stances over licensure exams

Controversy over disclosure of test items has centered on two issues: fairness and validity. The former issue relates to the need for freedom of information in a democratic society, especially if making that information public is in the public's best interest, while the latter relates to measurement issues and the potential threats to validity of test scores and their interpretation when items are disclosed to the public. Neither issue can be easily dismissed. Those in favor of fairness in testing claim that examinees should be allowed to refer to test content, answers, and other relevant materials. They contend that licensure tests should be regulated by social policies and undergo public scrutiny and evaluation. They prioritize on the principle of fairness, rather than on the validity of the tests, stating that the disclosure of test items would not undermine the reliability and validity of licensure. In addition, they contend that the credibility of tests can be guaranteed even if previously used test questions are disclosed, as long as these items are managed appropriately.

In contrast, opponents of disclosure argue that it will have a negative impact on test validity, thereby undermining the fairness of the tests. Retaining the standardized test format, they claim, provides all test-takers with a fair chance to do well, making the test into a more democratic tool (7, 8). They call into question the effectiveness and the potential benefit of making testing materials public, and demand clear empirical evidence to prove otherwise. In addition, they raise the question of whether test questions should be publicized continuously.

### Examples of regulation on test item disclosure

In the United States, item disclosure was first discussed at the state level. Between 1977 and 1983, approximately 90 bills were proposed from 28 states, including five at the federal House of Representatives. Of these, only New York and California passed their bills. These legislative actions were later known as *Truth-in-Testing* (TiT) *laws*. Despite being enacted in only two states, this legislation had a significant influence across the United States (9–13).

The California Senate Bill 2005, which passed in September 1978, mandated that actual test items and answers be made available to students, parents, and teachers. The law applies to all entrance exams for post-secondary or professional school admissions, exams taken by over 3,000 students yearly, including the Scholastic Aptitude Test (SAT) and the American College Test (ACT). Under the law, test agencies were required to file as a matter of public record reports that analyze the characteristics, limitations, and effectiveness of tests so that examinees can have access to information relevant to tests; agencies were also required to submit documents pertaining to the management of the tests and to related expenses. The New York bill, adopted in 1979, similarly stipulates the disclosure of detailed information relating to the enforcement and management of post-secondary admissions tests. Furthermore, test agencies are required to submit reports to the New York Commissioner of Education within 30 days after the test, and relevant materials need to be made available when requested by examinees. The law continues to generate controversy.

Similar bills were also proposed, but failed to pass in states including Florida, Maryland, Ohio, Texas, Colorado, Massachusetts, Pennsylvania, and New Jersey. In addition, two federal bills pertaining to the disclosure of test items were proposed but failed to pass in 1979: the Truth-in-Testing Act of 1979 (the Gibbons Bill) and the Educational Testing Act of 1979 (the Weiss Bill). The former involved disclosure of academic achievement test and occupational admissions test information, as well as school admissions test information, while the latter made provisions for more limited disclosure of materials concerned with tests.

### Legal actions by test agencies against mandated disclosure of test items

The mandated disclosure of test questions and answers stipulated by the New York legislation was debated following its passing. In 1979, the Association of American Medical Colleges (AAMC), which administers the Medical College Admissions Test (MCAT), filed an appeal against New York State, claiming that the disclosure of information about standardized admissions tests would violate its federal copyright (14). The AAMC stated that without an enforcement ordinance for non-disclosure of the MCAT, it would retract testing administration in New York. In 1980, the United States district court issued a preliminary order pertaining to the non-disclosure of test questions, but this did not lead to the revocation of the TiT law because the (federal) court observed that the issue was one of state division of power.

However, in 1988, the AAMC requested a permanent order that would invalidate the aforementioned law. After test items were made available to the public, scholars noted issues related to test difficulty. In response, testing agencies began to disseminate sensitivity reviews in place so that test bias could be controlled. Moreover, the expense of managing test questions were far less than expected, and the disclosure of test content boosted their income. Test agencies began to devote resources to developing and researching new test items. In particular, they prioritized test score equating to maintain the credibility of each test.

Still, the federal court filed a permanent order to prevent post-secondary admissions test information from being made available for public use, accepting the AAMC's appeal that disclosure would make it impossible for the Association to reuse its prior test items. The AAMC contended that disclosure provisions forced it to keep developing new items for examinations, resulting in a cumbersome financial loss and time.

## Effect of item disclosure on the sensitivity of the cut-off score

Passing scores on tests should be set in relation to minimum acceptable standards for practice, established by a national panel of content experts. Generally, international standards recommend against having a fixed cut score. For example, South Korea's Medical Licensure Examination sets its passing mark at 60% of the total possible marks, regardless of test characteristics (e.g., test difficulty) of each test form. As such, there is a high possibility that the test could misclassify some examinees due to the arbitrary cut score. This type of absolute standard can cause the proportion of candidates who are successful to change the difficulty level of each test form; difficult test forms can yield fewer successful candidates and vice versa, thereby undermining the validity of the test's cut-off score. In contrast, a *criterion-referenced standard setting*, which considers difficulty and format, enables experts from various fields to set minimum requirements for passing, and therefore, the passing mark could potentially change for each test administration.

**Fig. 1:**
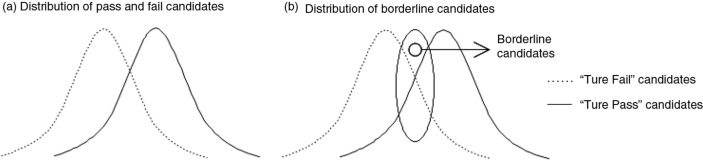
Virtual distribution of pass and fail candidates. Dotted line (…) indicates ‘true fail’ candidates; solid line (–) indicates ‘true pass’ candidates.

**Fig. 2:**
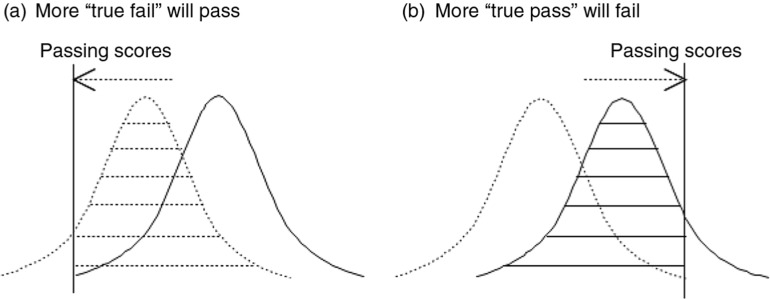
Setting passing scores.

In determining cut-off scores, the possibility of measurement error should be considered. Figure 1 shows that the results of borderline candidates can differ from what was expected when the cut score was set. Figure 2 demonstrates that if the cut-off score is lowered (i.e., line is placed to the left), a number of examinees with insufficient qualifications (‘true fail’ candidates) will pass the exam, while if the cut line is placed on the right (a higher threshold), candidates who might have qualified under other conditions (‘true pass’ candidates) will fail the test.

Since the disclosure of test items could result in changes in the test's difficulty and characteristics in the following years, it is imperative to have a comprehensive plan for considering errors related to measurement and selection of test items when designing licensure examination. Such a plan should consider borderline candidates at the initial stage of test design, so that more consistent measurement range could be presented, preventing unfair passing or failure of such candidates (see Fig. 1b). In the United States, there has been a body of research on errors in determining cut scores and maintaining consistency of cut lines across tests. For example, the United State Medical Licensure Examination (USMLE), administered by the National Board of Medical Examiners (NBME), has turned to the Angoff and Hofstee methods in determining cut score (15). The former approach involves a group of subject matter experts setting minimal qualifying requirements (in terms of test score) for a successful candidate, who will later be certified as a medical doctor. The Board's opinion serves as a foundation for the evaluation of each test item and a reference for determining cut score. The latter method asks judges to determine minimum/maximum acceptable scores, along with minimum/maximum fail rates, which serve as the key parameters in determining the final cut score. The NBME continues to re-evaluate its cut score. Recently, Step 1 cut-off score was adjusted from 188 to 192 (out of 300) in January 2014.

## Utility of test item disclosure

Hale, Angelis, and Thibodeau who examined the results of the Test of English as a Foreign Language (TOEFL), found that item disclosure could affect future tests considerably (16). In particular, they claimed that examinees would perform better if taking a test containing previously disclosed items, since test-takers could then potentially choose correct answers from recall, without fully understanding the question. This could undermine the validity and credibility of the test. In contrast, Stricker argued that the disclosure of previous test items had little impact on examinees’ performance, citing the results of the SAT after the move to item disclosure (17). In this study, Stricker randomly selected examinees and had them retake the test; the examinees’ performance did not show any significant improvement, and therefore, concluded that disclosure did not affect the performance of test-takers. Similarly, using data from the Medical Council of Canada Qualifying Examination (MCCQE), Wood found that test-takers did not benefit from disclosure (18).

However, opponents of item disclosure mainly point to the possibility of equating error in the process of test equating and the application of equated test scores. The purpose of test equating is to determine what scores on different versions of an exam indicate comparable achievement. A test designed without an appropriate process of score equating will not factor in variables such as the difficulty of items and changes in test environment. Based on an analysis on the influence of item disclosure, Gilmer found that continuous disclosure of test items would result in higher pass rates regardless of examinees’ performance, which would lead to unfair benefits for those who share the previous test items (19).

As noted above, the key to administering professional licensure exams is to maintain acceptable levels of validity and credibility for the test to serve its gate keeping function and the public to be provided with appropriate services. Examination alone cannot guarantee this, but licensure tests could serve as an important measurement for investigating whether a candidate could provide safe and efficient services to consumers (20).

The *Standards for Educational and Psychological Testing* states that the ultimate goal of issuing licensure and certification is to protect the public's rights (6). Disclosure of test items could pose a threat to undermine this goal by allowing unqualified applicants to pass the test. To date, however, there is insufficient empirical research on the usefulness and impact of test disclosure, particularly large-scale studies that can help draw inferences and policy implications. Therefore, it is high time to conduct comprehensive studies on item disclosure to protect the public's rights and to improve the quality of services. Additional studies are underway to examine the empirical impact of item disclosure on psychometric test characteristics, passing levels, and perceptions of test takers – to help inform policies and contribute to findings on item disclosure for licensing examinations.

## Conclusion

This study provides a historical overview of item disclosure through the perspective of TiT legislation in the United States, synthesizes existing literature, and discusses implications of item disclosure in the context of international testing standards. As context, guidelines from the *Standards for Educational and Psychological Testing* are described, as it provides fair and credible standards for designing and managing exam items; however, some countries have yet to adopt appropriate guidelines.

While there are some noted advantages of test item disclosure – fulfilling the public's need to know on test information, allowing test-takers to check the right answer and verify whether test results were processed properly, and enabling experts to examine test items for potential bias – it may lead to more drawbacks and problems. Most testing institutions and experts in the United States and Europe maintain a negative stance on disclosure, citing possible declines in test validity, as well as the possibility that disclosure can alter test characteristics, making it impossible to conduct comparative analyses of each test based on test equating. Moreover, item disclosure hinders the reuse of test items; and as such, developing new test items every year would require considerable financial and human resources. Due to these limitations and constraints, a broader and careful consideration toward item disclosure in medical licensing examination is needed, one that adheres to international testing standards, optimizes test validity, and most of all, ensures the safety of the public by licensing only qualified individuals to enter the health professions arena.

## References

[1] Leary LF, Dorans NJ (1985). Implications for altering the context in which test items appear: a historical perspective on an immediate concern. Rev Educ Res.

[2] Powers DE, Fowles ME (1998). Effects of pre-examination disclosure of essay topics. Appl Psychol Meas.

[3] Veerkamp WJJ, Glas CAW (2000). Detection of known items in adaptive testing with a statistical quality control method. J Educ Behav Stat.

[4] Meijer RR (2003). Diagnosing item score patterns on a test using item response theory-based person-fit statistics. Appl Psychol Meas.

[5] Hendrawan I, Glas CAW, Meijer RJ (2005). The effect of person misfit on classification decisions. Appl Psychol Meas.

[6] American Educational Research Association (AERA), American Psychological Association (APA), National Council on Measurement in Education (NCME) (2014). Standards for educational and psychological testing.

[7] Bersoff DN (1981). Testing and the law. Am Psychol.

[8] Bower DR (1987). Test disclosure and bias issues: the New York experience. Federal Bull.

[9] Florio DH (1979). Congress watch: truth in testing, funding cuts, and the Department of Education. Educ Res.

[10] Greer DG (1984). “Truth-in-testing legislation,” an analysis of political and legal consequence, and prospects (Monograph No. 83–6).

[11] Greer DG (1984). Legal issues in truth-in-testing legislation. Rev High Educ.

[12] Messick S (1981). Evidence and ethics in the evaluation of tests. Educ Res.

[13] Dorans NJ (2012). The contestant perspective on taking tests: emanations from the statue within. Educ Meas.

[14] Espinoza LG (1993). The LSAT: narratives and bias. J Gend Law.

[15] Cizek GJ (2012). Setting performance standards: foundations, methods, and innovations.

[16] Hale GA, Angelis PJ, Thibodeau LA (1980). Effects of item disclosure on TOEFL performance. ETS Research Report 80-34.

[17] Stricker LJ (1984). Test disclosure and retest performance on the SAT. Appl Psychol Meas.

[18] Wood TJ (2009). The effect of reused questions on repeat examinees. Adv Health Sci Educ.

[19] Gilmer JS (1989). The effects of test disclosure on equated scores and pass rates. Appl Psychol Meas.

[20] California Department of Consumer Affairs (1983). What a licensing board member needs to know about testing.

